# Small cell neuroendocrine carcinoma expressing alpha fetoprotein in the endometrium

**DOI:** 10.1515/biol-2021-0122

**Published:** 2021-11-09

**Authors:** Weiwei Hou, Bin Zhou, Gang Hou, Yu Pang, Jing Sang, Ning Li

**Affiliations:** Department of Pathology, Tai’an Central Hospital, No. 29, Longtan Road, Tai’an District, Tai’an 271000, China; Traditional Chinese Medicine Gynecology Department, Tai’an Central Hospital, No. 29, Longtan Road, Tai’an District, Tai’an 271000, China

**Keywords:** alpha-fetoprotein, small cell neuroendocrine carcinoma, immunohistochemistry, endometrium, vaginal bleeding

## Abstract

Rare small cell neuroendocrine carcinoma (SCNEC) cases showed alpha fetoprotein (AFP) expression in the endometrium. In this study, we reported a case of uterine SCNEC expressing AFP. In addition, a literature review was performed to investigate the potential mechanism and the clinicopathological features of SCNEC to provide clinical guidance. A 65-year-old female was referred to our hospital due to vaginal bleeding for 1 month in November 2020. The clinical features were summarized. After total hysterectomy and removal of bilateral appendages, the histological examination and immunohistochemistry examination were performed. Histological findings showed that the cancer cells were arranged in a nest-like pattern distributed in a lamellar manner. The smooth muscles of the uterus were invaded by cancer cells. Cancer cells were relatively consistent in size. Small glandular duct-like and rosettes-like structures were distinguished, together with necrotic tissues. The deep staining showed that the amount of cytoplasm was lower in the nucleus. Partial cancer cells had small nucleolus with an irregular profile. There were some mitotic figures. Immunohistochemistry examination indicated that there was a diffuse expression of CK, Syn, CgA, CD56, CK8/18, P16, AFP, HepPar-1, Glypican-3, and Ki67 (90%). In this case, we reported a SCNEC patient expressing AFP, Glypican-3, and HepPar-1.

## 1 Introduction

Small cell neuroendocrine carcinoma (SCNEC) is a malignance usually occurring in the pulmonary tissues [1]. Also, it may occur in the gastrointestinal tract, female reproductive system, head and neck, as well as mammary gland [2]. For the patients with female reproductive system involvement, SCNEC commonly involves the cervix, while rare cases show endometrial involvement with a prevalence of merely 0.8% among all endometrial carcinomas [3].

SCNEC is featured by a high-grade malignancy, strong invasion, distal metastasis, as well as a poor prognosis [4,5]. Rare SCNEC cases showed expression of alpha fetoprotein (AFP) after a literature review [6]. In this study, we reported a case of uterine SCNEC expressing AFP. In addition, a literature review was performed to investigate the potential mechanism and the clinicopathological features of SCNEC in order to provide clinical guidance. We present the following article in accordance with the STROBE reporting checklist.

## 2 Materials and methods

### 2.1 Clinical data

A 65-year-old female was referred to our hospital due to vaginal bleeding for 1 month. She showed no abdominal pain and distension. She underwent a hysteroscopy in a local hospital. Diagnostic curettage-based pathology indicated malignance combined with necrosis. On this basis, the patient was highly suspected of poorly differentiated carcinoma or endometrial stromal sarcoma. On physical examination after admitting to our hospital, there were dilation and hydrops in the uterine cavity after the abdominal plain scan. Additionally, irregular lumps with equal density were noticed in the left posterior uterine wall, together with prominence (3.7 cm × 2.7 cm) in the urine cavity, presenting cauliflower-like changes on the surface and heterogeneous enhancement. A CT scan demonstrated occupying lesions in the uterine cavity, and then endometrium malignancy was considered. No abnormality was seen in other organs. Laboratory examination findings were as follows: AFP, 975.30 ng/mL (normal range: 0–10); C-reactive protein (CRP), 165.89 mg/L (normal range: 0.00–9.00); and D-dimer, 2.23 mg/L DDU (normal range: 0–1.00). The patient underwent a total hysterectomy and removal of bilateral appendages. A pathological examination was performed after the surgery.


**Informed consent:** Informed consent has been obtained from all individuals included in this study.
**Ethical approval:** The research related to human use complied with all relevant national regulations, institutional policies and was in accordance with the tenets of the Helsinki Declaration, and has been approved by the Ethics Committee of Tai’an Central Hospital (No.: WD-0019).

### 2.2 Methods

All the samples were fixed in 10% neutral formalin, followed by embedding in paraffin. Sections (4 μm) were stained by hematoxylin and eosin. The antibodies used were as follows: PAN-cytokeratin (CK, Catalog number: RAB-0050; 1:2,000), synaptophysin (Syn, Catalog number: MAB-0742; 1:1,000), chromogranin A (CgA, Catalog number: MAB-0707; 1:1,000), neural cell adhesion molecule (CD56, Catalog number: MAB-0743; 1:2,000), Glypican-3 (Catalog number: MAB-0617; 1:2,000), cytokeratin 8/18 (CK8/18, Catalog number: MAB-0650; 1:2,000), CD10 (Catalog number: MAB-0668; 1:1,000), estrogen receptor (ER, Catalog number: MAB-0062; 1:1,000), progesterone receptor (PR, Catalog number: MAB-0675; 1:2,000), P53 (Catalog number: MAB-0674; 1:2,000), cyclin dependent kinase-4 (P16, Catalog number: MAB-0673; 1:2,000), cytokeratin-7 (CK7, Catalog number: MAB-0828; 1:1,000), as well as Ki-67 (Catalog number: RMA-0542; 1:1,000). All reagents were purchased from Maixin Biotech (Fuzhou, China). The pathological images were analyzed using CaseViewer software.

## 3 Results

### 3.1 General conditions

**Figure 1:**
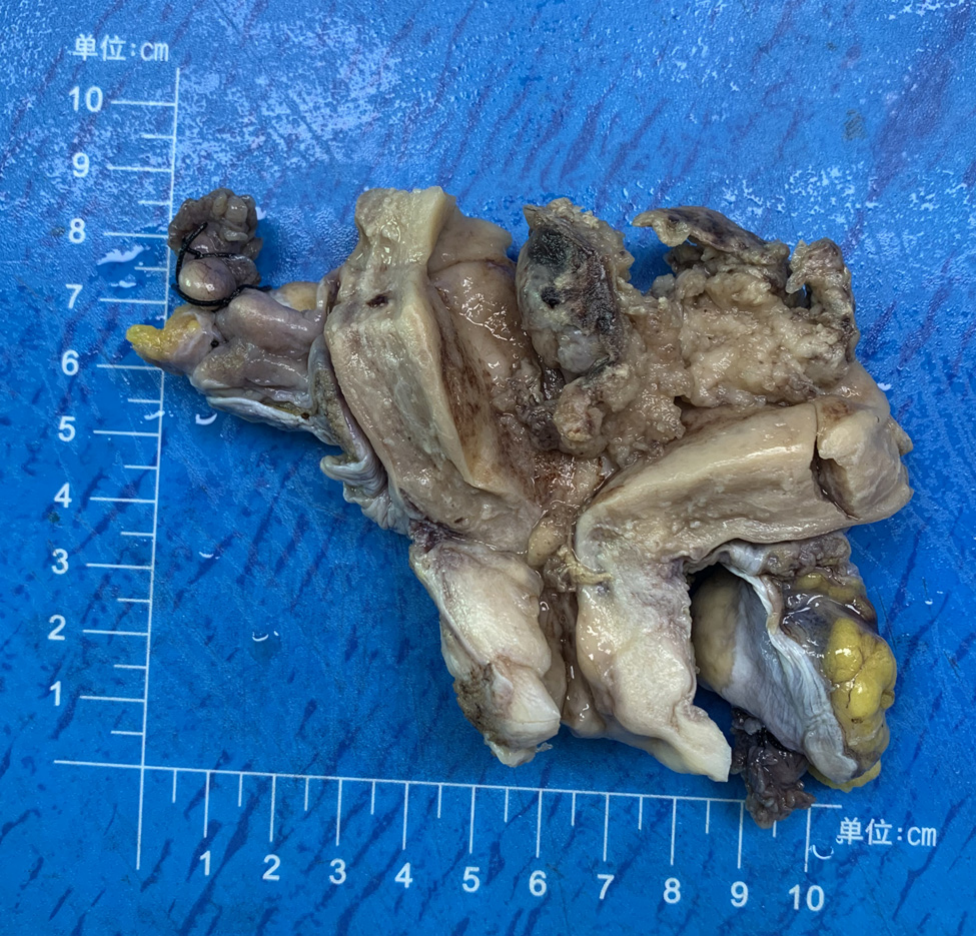
A mass in the left horn of the uterus with a size of 4 cm.

The uterus with a size of 9 cm × 8 cm × 2 cm had been dissected along the anterior wall. The cervical canal showed a length of 2.5 cm, with a diameter of about 2.5 cm. The mucous membrane at the external orifice was smooth, and the uterine depth was 7 cm. The thickness of the muscular layer was in a range of 1.5–2.0 cm. There was a tumor mass (4 cm × 3.5 cm × 2.5 cm) near the left horn of the uterus. This incisal surface was in a grayish-white color mixed with red color in a hard texture. The depth of invasion was less than half of the myometrium. The bilateral appendix was normal in structure (Figure 1).

### 3.2 Histological findings

**Figure 2:**
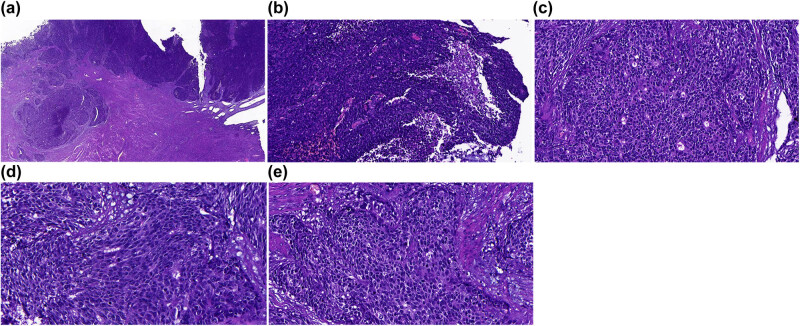
HE staining of the samples. (a) Cancer cells were arranged in a nest or lamellar pattern. Infiltrative growth was noticed in the myometrium. (b) Necrosis in the cancer tissues. (c) Small glandular duct-like and rosettes-like structures. (d) Multiple mitotic phases. (e) Cancer cells with less cytoplasm, and deep staining in nucleus. Part of the cancer cells showed a small nucleus. Magnifications of the images were as follows: (a) 40×; (b and c) 200×; (d and e) 400×.

Under a magnification of 40×, the cancer cells were arranged in a nest-like pattern, distributed in a lamellar manner. The smooth muscles of the uterus were invaded by cancer cells (Figure 2a). Cancer cells were relatively consistent in size under a magnification of 200×. Small glandular duct-like and rosette-like structures were distinguished with necrotic tissues (Figure 2b and c). The cytoplasm was less in amount with deep staining in the nucleus under a magnification of 400×. Partial cancer cells showed small nucleolus with an irregular profile. There were some mitotic figures (15 per high power, Figure 2d and e).

### 3.3 Immunophenotypes

**Figure 3:**
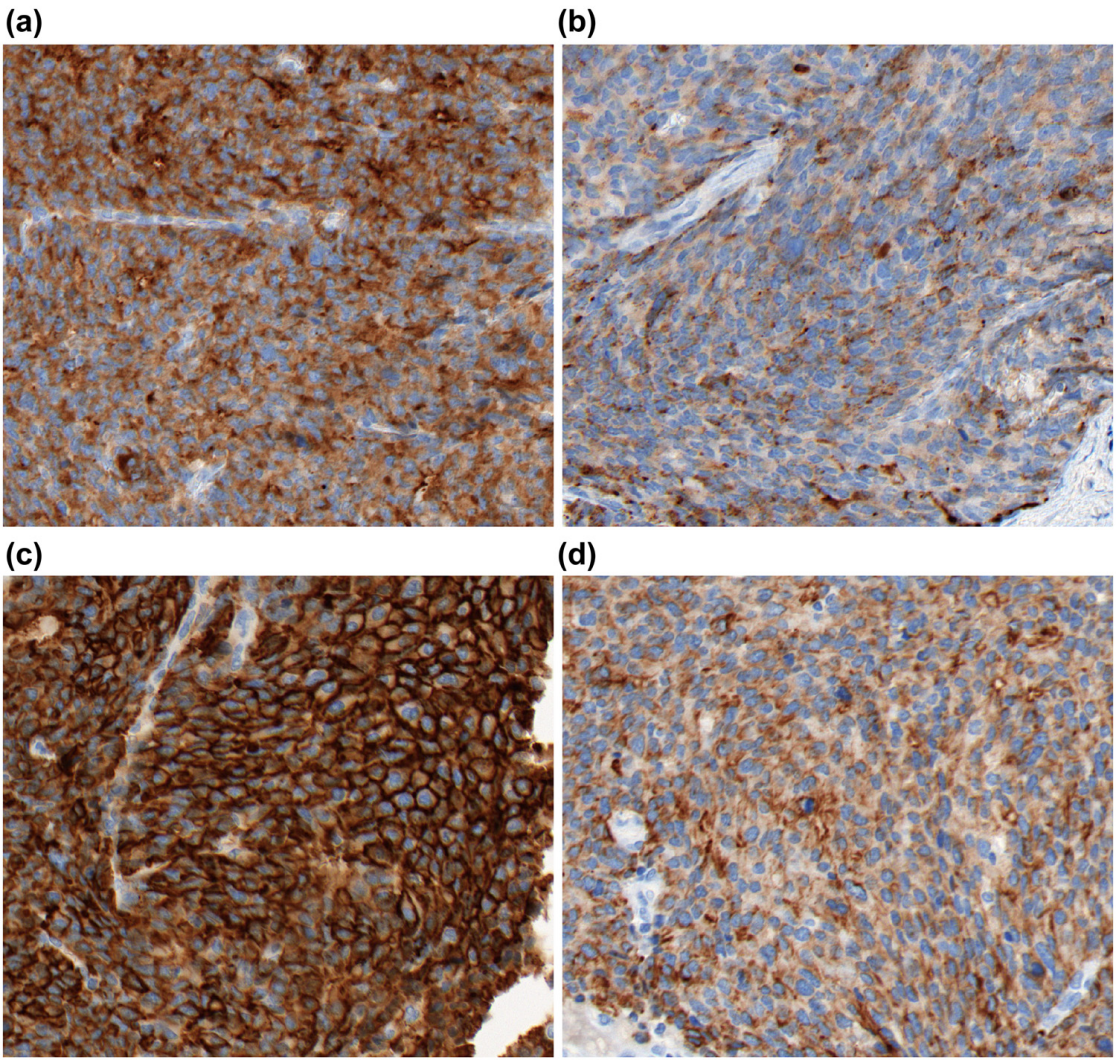
Immunohistochemistry findings. (a) The cytoplasm in cancer cells was sparse, with the nucleus stained in a deep color. There was a small nucleus in partial cancer cells. (b–d) Expression of Syn, CgA, Glypican-3, and AFP in cancer cells. The images were observed under a magnification of 400×.

There was positivity for the immunophenotypes of cancer cells for CK, AFP, Syn, CgA, CD56, HepPar-1, Glypican-3, CK8/18, P16, and P53 (Figure 3a–d). The expression of CK7, ER, PR, Vimentin, and CD10 was negative. The ki-67 index was approximately 90%.

### 3.4 Final diagnosis

Based on these findings mentioned above, the patient was finally diagnosed with SCNEC expressing AFP in the endometrium.

### 3.5 Follow-up

The patient received no treatment previously. She was followed up for 2 months after surgery. The serum AFP showed a decline after treatment, which was in the normal range.

## 4 Discussion

SCNEC is a rare malignancy featured by a high possibility of metastasis and a poor prognosis. To date, specific therapeutic options are still lacking for it [7]. Nowadays, there are some hypotheses for the histological origin of SCNEC. The origins were supposed to evolve from endometrial neuroendocrine cells, multifunctional and multiple differentiation potential stem cells in the endometrium, differentiated neuroendocrine cells of endometrial adenocarcinoma, differentiated tumor cells of endometrial adenocarcinoma in the presence of living conditions and endocrine factors, as well as the multidirectional differentiation of the Mullerian ducts such as differentiating into neuroendocrine cells. To the best of our knowledge, rare SCNEC cases expressed AFP [8]. In this study, we reported a SCNEC case expressing AFP and summarized the clinical and pathological features as well as the treatment and outcome.

SCNEC is commonly seen in females of perimenopausal or post-menopause periods. The common symptoms include irregular vaginal bleeding. As previously described [9], patients with SCNEC were mainly manifested as a dystopia, membranous glomerulonephritis, and Cushing syndrome. Partial patients showed a slight elevation in CA125 and NSE. The cancer cells showed a strong invasion capacity, with rapid progression. The lesions were highly malignant, with a high possibility of metastasis to lung, bones, and brain tissues [10].

The lesions of SCNEC were featured by cauliflower-like or polypoid-like mass in the uterine cavity, together with invasive growth in the myometrium. The incisal section was white, with a hard texture. Part of the lesions showed fish-meat-like changes, frequently combined with bleeding and necrosis. The cancer cells were distributed in a nest-like, lamellar and rosette-like pattern. The cellular size was not large, which was relatively consistent in size. The cytoplasm was not much, and the nucleus was stained strongly. Partial cancer cells showed a small nucleus with an irregular profile. There were some mitotic figures combined with endometrial adenocarcinoma or squamous cell carcinoma. In a previous study, Van Hoeven et al. proposed the diagnostic standards for SCNEC as follows: (i) with definite evidence for primary lesions in endometrium; (ii) consisted of cancer cells of similar sizes, in a dense or lamellar growth pattern, with other cancer components; and (iii) expressing at least one neuroendocrine markers in the immunohistochemistry. To date, the commonly utilized immunohistochemistry markers included NSE, CgA, Syn, CD56, P53, P16, and Ki67 in a range of 60–90% [11]. In a previous study, there was a high CgA, Syn, and NSE expression in SCNEC patients [12]. Therefore, the diagnosis of SCNEC relied on the combination of histopathological analysis and immunohistochemistry. In this case, there was expression of Syn, CgA, and CD56, together with AFP, HepPar-1, Glypican-3, CK8/18, and Ki67 (90%). Finally, the patient was diagnosed with SCNEC expressing AFP.

In clinical practice, SCNEC patients with endometrial AFP expression should be differentially diagnosed from the other types of cancers. For example, as in this case, the immunohistochemistry findings were positive for AFP, Glypican-3, and HepPar-1, and it should be distinguished from hepatoid adenocarcinoma of the endometrium. In histology and morphology, the hepatoid adenocarcinoma of the endometrium was similar to hepatocellular liver cancer. The cancer tissues could specifically express AFP, Glypican-3, and HepPar-1; however, they did not express NSE, Syn, and CD56. In addition, it should be distinguished from small cell squamous cell carcinoma, which showed a small and round profile in cancer cells, together with eosin staining in the cytoplasm and deep staining in the nucleus. Occasionally, there might be single-cell hornification. The immunohistochemistry for P63 was positive, while the neuroendocrine markers were all negative. Moreover, it should be distinguished from the poorly differentiated endometrioid carcinoma. The majority (90%) of these patients showed vaginal bleeding, and the cancer cells showed solid and lamellar growth, with none or less an adenoid structure. The cellular differentiation was poor, and there were multiple mitotic phases. There was a strong expression of ER and PR, and the neuroendocrine markers were all negative. Furthermore, SCNEC should be further distinguished from endometrial stromal sarcoma in which the cells were arranged in a lamellar or cluster pattern. The cancer cells were in a short and spindle shape or an oval shape, which distributed along the vessels. In addition, there was collagenization and branch-like vessels. The immunohistochemistry for CD10 was positive, and the neuroendocrine markers were negative. Finally, attention should be paid to the differential diagnosis of metastatic small cell carcinoma. The small cell carcinoma usually occurs in the lungs, while in the female population, it usually involves the cervix and/or ovary gland. Furthermore, SCNEC should be differentially diagnosed with carcinosarcoma, designated as a Mullerian mixed tumor that usually occurs in the aged female. The epithelial component is usually high-grade carcinoma, mainly featured in serous carcinoma and endometrioid carcinoma. The interstitial component consists of homologous and heterogenous types, such as the high-grade endometrial stromal sarcoma and the rhabdomyosarcoma. The immunohistochemistry contributed to the confirmation of the high-grade carcinoma and interstitial components in the carcinosarcoma. The malignant epithelial cells usually expressed p53, p16, and PTEN, while the malignant interstitial cells expressed CD10 and Vimentin rather than Syn, CgA, and CD56. In this case, there were no lesions in other sites after the whole body imaging scan except endometrium, and there were no aberrant changes in the cervix and ovary gland.

At the early stage, there might be local infiltration and lymphatic metastasis. Most cases showed survival of less than 1 year. To date, there is still a lack of SCNEC cases, and its treatment efficiency is still not clear due to a lack of clinical data and treatment standards. To date, the treatment of SCNEC is highly dependent on surgery, chemotherapy, and radiotherapy, and lactate dehydrogenase plays an important role in the evaluation of its prognosis [13]. The surgery scale follows that of the endometrioid carcinoma, including total hysterectomy, bilateral adnexectomy, intrapelvic lymph node dissection, para-abdominal aorta lymph node dissection, and greater omentum resection [14]. In addition, the regimens for chemotherapy and radiotherapy are in accordance with the small cell lung cancer, involving the combination of VP-16 and platinum-based chemotherapy [7,15,16,17].

Indeed, there are some limitations to this study. We could not find out the potential relationship between AFP expression and SCNEC in this study as this is a case report. In the future, animal models should be utilized to investigate the potential relationship.

In summary, SCNEC is rare cancer involving the reproductive system in the female population, with a strong invasion and a high possibility of metastasis. In this case, we reported a SCNEC patient expressing AFP, Glypican-3, and HepPar-1. In addition, the AFP was higher than the normal range before treatment. However, it recovered to the normal range about 2 months after follow-up.
